# Genomic Bayesian functional regression models with interactions for predicting wheat grain yield using hyper-spectral image data

**DOI:** 10.1186/s13007-017-0212-4

**Published:** 2017-07-27

**Authors:** Abelardo Montesinos-López, Osval A. Montesinos-López, Jaime Cuevas, Walter A. Mata-López, Juan Burgueño, Sushismita Mondal, Julio Huerta, Ravi Singh, Enrique Autrique, Lorena González-Pérez, José Crossa

**Affiliations:** 1 Departamento de Matemáticas, Centro Universitario de Ciencias Exactas e Ingenierías (CUCEI), Universidad de Guadalajara, 44430 Guadalajara, Jalisco Mexico; 2 Facultad de Telemática, Universidad de Colima, Colima, Mexico; 3 Universidad de Quintana Roo, Chetumal, Quintana Roo Mexico; 4 Facultad Ingeniería Mecánica y Eléctrica, Universidad de Colima, Colima, Mexico; 5 International Maize and Wheat Improvement Center (CIMMYT), Apdo. Postal 6-641, 06600 Mexico, D.F. Mexico

**Keywords:** Hyper-spectral data, Genomic information, Genotype × environment interaction, Band × environment interaction, Vegetation indices, Prediction accuracy, Bayesian functional regression, Spline regression, Fourier regression

## Abstract

**Background:**

Modern agriculture uses hyperspectral cameras that provide hundreds of reflectance data at discrete narrow bands in many environments. These bands often cover the whole visible light spectrum and part of the infrared and ultraviolet light spectra. With the bands, vegetation indices are constructed for predicting agronomically important traits such as grain yield and biomass. However, since vegetation indices only use some wavelengths (referred to as bands), we propose using all bands simultaneously as predictor variables for the primary trait grain yield; results of several multi-environment maize (Aguate et al. in Crop Sci 57(5):1–8, 2017) and wheat (Montesinos-López et al. in Plant Methods 13(4):1–23, 2017) breeding trials indicated that using all bands produced better prediction accuracy than vegetation indices. However, until now, these prediction models have not accounted for the effects of genotype × environment (G × E) and band × environment (B × E) interactions incorporating genomic or pedigree information.

**Results:**

In this study, we propose Bayesian functional regression models that take into account all available bands, genomic or pedigree information, the main effects of lines and environments, as well as G × E and B × E interaction effects. The data set used is comprised of 976 wheat lines evaluated for grain yield in three environments (Drought, Irrigated and Reduced Irrigation). The reflectance data were measured in 250 discrete narrow bands ranging from 392 to 851 nm (nm). The proposed Bayesian functional regression models were implemented using two types of basis: B-splines and Fourier. Results of the proposed Bayesian functional regression models, including all the wavelengths for predicting grain yield, were compared with results from conventional models with and without bands.

**Conclusions:**

We observed that the models with B × E interaction terms were the most accurate models, whereas the functional regression models (with B-splines and Fourier basis) and the conventional models performed similarly in terms of prediction accuracy. However, the functional regression models are more parsimonious and computationally more efficient because the number of beta coefficients to be estimated is 21 (number of basis), rather than estimating the 250 regression coefficients for all bands. In this study adding pedigree or genomic information did not increase prediction accuracy.

## Background

In plant breeding, a branch of agriculture, plant phenotyping has become financially expensive when evaluating complex traits like grain yield in large numbers of selection candidates [8, 11]. However, since the cost of sensors, aeronautics, high performance computing and high-resolution cameras has decreased significantly, plant breeders now have greater capacity to measure electromagnetic energy at varying wavelengths that interact with different parts of the growing plant. For this reason, the use of low-cost, efficient, high-throughput plant phenotyping platforms (HTPP) [2] has dramatically increased. By means of HTPP, it is possible to collect many low-cost phenotypes on large numbers of breeding individuals at different stages of plant growth under different environmental conditions. There is a consensus that collecting many phenotypes of primary and secondary traits at an early stage of plant growth could be of great value for reducing evaluation time and cost, while dramatically increasing selection intensity and prediction accuracy and, consequently, the response to selection [16].

One important characteristic of current HTPP is their capability to non-destructively capture plant traits. This allows time-series measurements that are necessary to follow the progression of growth and stress on individual plants. Eliminating destructive measurements also increases the experimental capacity of genotypes, treatments, and biological replicates by reducing the required replicated sampling sets. High-throughput image-based phenotyping is defined as a technology that can generate images of hundreds of plants per day. With a population in the hundreds, it is possible to analyze mutant populations, detect QTLs, discover gene × environment associations [6], and increase the prediction accuracy of the primary trait (grain yield) by using pedigree or genomic information [16].

The main goal of plant imaging is to measure the physiological growth, developmental, and other phenotypic properties of plants through automated processes using digital camera technology to collect reflectance data of electromagnetic energy at different wavelengths. The collected reflectance data are then used to predict plant physiological or agronomic traits such as grain yield. There are two main approaches for using reflectance data: (1) use partial reflectance data, summarize them in scores called spectral vegetative indices (VI) and then use the VI as predictor variables for primary traits; and (2) use all reflectance data simultaneously to predict the primary trait of interest. Despite some successful applications of the first approach, it has been criticized because it does not consider all the spectral bands from the hyperspectral sensors and because most VI tend to be species-specific. This means they are not robust when applied across different species that have different canopy architectures and leaf structures because they use only a fraction of the available information on the measured wavelengths. Using all bands is more robust and gives better prediction accuracy of primary traits (e.g., grain yield) than VI, as already reported [1, 13].

Recently secondary traits have been incorporated into vegetative indices (e.g., canopy temperature and normalized difference vegetation index) using multivariate pedigree and genomic prediction models by means of random regression models [18]. The authors showed that within each environment, the best linear unbiased predictions (BLUP) of secondary traits used in the multivariate prediction model substantially improved (by 70%, on average) the prediction of primary trait grain yield.

However, to the best of our knowledge, until now no studies have been conducted on HTPP data analyses that take into account not only all the reflectance bands measured in different environments, but also the genomic (and pedigree) information and the interactions between genotype and environment (genotype × environment, G × E) and between the band and environment (band × environment, B × E). In plant breeding, there is enough evidence that when the genomic (or pedigree) information and the G × E are taken into account, the models do better in terms of prediction accuracy. Also, it is well documented that if the same genotype is exposed to different environments, significant differences in the phenotype of plants, animals or any living organism can be expected even if the original individuals had similar genetic composition. One of the first scientists to note that the effect of genes (G) on phenotype could be modified by the environment (E) was Garrod [9]. For this reason, Turesson [19] pointed out that the environment often influences plant development and that the presence of a particular variety in a specific location is not just a chance occurrence; rather, the variety’s peculiar characteristics are attributable to the effect of the environment on the expression and function of the genes influencing the trait.

To better illustrate the importance of considering the G × E interaction term and its effects on prediction accuracy, we provide some examples. Most of the time in genomic-enabled prediction, multi-environment models with G × E have better prediction accuracy than single-environment models. For example, using wheat data, Jarquin et al. [10] found that the prediction accuracy of models including interaction terms was substantially higher (17–34%) than that of models based on main effects only. For a maize ordinal data set, Montesinos-López et al. [12] found that compared to models based only on main effects, models that included G × E achieved gains of 9–14% in prediction accuracy. Using wheat data, Cuevas et al. [3] found that models with the G × E term were up to 60–68% better than the corresponding single-environment models. However, in the HTPP context, no models have been developed that include G × E as well as genomic or pedigree information; furthermore, no research has been conducted and published on assessing the possible effect of B × E on predicting the primary trait.

However, taking into account both interaction terms also increases the computational cost of implementing this model since the dimensionality of the predictor grows in proportion to the number of environments and bands. For this reason, Montesinos-López et al. [13] proposed using functional data analysis to help reduce the computational cost by reducing the dimensionality of the bands. Functional data analysis is a branch of statistics that studies and analyzes information contained in curves, surfaces, or any element that varies over a continuum, usually time. In its most general form, within a functional data framework, each sample element is considered to be a function. In general, any observation that varies on a continuum can be taken for functional data, from an electrocardiogram to urban temperatures. In practice, these events are measured by machines that take samples of a certain random variable at different moments in time within a certain range $(t_{\text{min} } , t_{\text{max} } )$. The physical continuum space over which these functions are defined (in addition to time) are wavelength, spatial location, age, etc. This mean that the data used for functional data analysis are repeated measures since for each individual are measured a sample of points in the range of time $(t_{\text{min} } , t_{\text{max} } )$ or wavelength and for each individual we obtain a curve which is approximated with some functions (B-slines, Fourier, etc.) that reduce the dimensionality of the original data point measured for each individual.

Therefore, based on the previous results, the main objectives of this research are: (1) to propose genomic Bayesian functional regression models that take into account the main effects of environment and genotype, all the available reflectance wavelength data, genomic or pedigree information, and the interaction terms (G × E and B × E) for predicting the primary trait grain yield; (2) to compare the prediction accuracy of models that include genomic (or pedigree) information versus those that do not; (3) to compare the prediction accuracy of models that include interaction terms versus those that do not; (4) to compare the prediction accuracy and implementation time of Bayesian functional regression models versus conventional Bayesian models that are not in the functional regression category; and (5) to identify models that have the best prediction performance and identify time-points of plant growth before harvesting from which accurate predictions of wheat grain yield can be obtained.

To illustrate the use of the proposed genomic Bayesian functional regression models and achieve the five objectives of this study, we used part of the data set employed by Montesinos-López et al. [13], which is comprised of 976 wheat lines from the CIMMYT Global Wheat Program that were evaluated for grain yield in three contrasting environments in Cd. Obregon, Mexico (Drought, Irrigated and Reduced Irrigation). A total of 250 wavelengths were measured at nine different time-points of crop growth (1–9). The original data set has 5 environments but the phenotypic information of three environments were almost identical with a correlation greater than 0.97. For this reason we only work with the information of three environments, also the original data set has 1170 wheat lines, but pedigree (relationship matrix **A**) and genomic relationship matrix (**G**) information was only available for only 976 wheat lines.

## Methods

### Phenotypic field trial data and high-throughput phenotypic data

A detailed description of the data used in this study can be found in Montesinos-López et al. [13], where the authors present several functional regression models for predicting grain yield using hyperspectral image data in each environment. In this study, we only used data from three environments—Drought, Irrigated, and Reduced Irrigation—and 976 lines of the original 1170 wheat lines from the CIMMYT Global Wheat Program [13]. The experimental design used was an alpha-lattice with three replicates and six incomplete blocks of size five for each replicate; two checks were included in each of the trails; these checks were part of the first stage analyses but not included in the genomic prediction model. This design was used in each of the 39 trials implemented in each environment, with 30 lines included in each trial. Traits grain yield (GY) and days to heading (DH) were measured in each line, but only GY was analyzed in this study. Planting dates in the three environments were December 1–5, 2014. The bands were measured on nine different dates (January 10, 2015, January 17, 2015, January 30, 2015, February 7, 2015, February 14, 2015, February 19, 2015, February 27, 2015, March 11, 2015 and March 17, 2015), which we call time-points (1, 2, 3,…, 9, respectively) using 250 discrete narrow wavelengths. In each plot for each line and at each time-point, 250 wavelengths $\lambda_{1} , \ldots \lambda_{250}$ from 392 to 851 nm were measured. The $k$ th discretized spectrometric curve is given by $x_{1} \left( {\lambda_{1} } \right), \ldots ,x_{250} \left( {\lambda_{250} } \right)$. We used the notation $x\left( {780} \right)$ without subscripts to denote the response of the band measured at 780 wavelength, $x\left( {670} \right)$ to denote the response of the band measured at 670 wavelength, and so on.

### Genotypic and pedigree data

Genotyping-by-sequencing (GBS) was used for genome-wide genotyping and single nucleotide polymorphisms were called across the lines using the TASSEL GBS pipeline anchored to the genome assembly of Chinese Spring. Single nucleotide polymorphisms were extracted and 34,900 GBS markers were filtered so that markers with more that 30% missing were deleted. Next, missing markers were imputed with the marker mean. Then each marker frequency is computed and markers with less than 0.05 minor allele frequency were removed. After this a total of only 1448 GBS markers remained after marker filtering and editing. A pedigree was used to devive the additive relationship matrix (**A**) among the wheat lines [16]; the entries of matrix **A** equal twice the coefficient of parentage between pairs of lines.

### Statistical models

Since the experiments were performed in three environments under an alpha-lattice experimental design with three replicates, 39 trials and six blocks in each replicate, the model proposed is

1 $$y_{ijklm} = E_{i} + L_{j} + LE_{ij} + r_{{k\left( {l,i} \right)}} + t_{l\left( i \right)} + b_{{m\left( {l,k,i} \right)}} + \epsilon_{ijklm} ,$$

where $y_{ijkl}$ is the response variable (GY or wavelength measurements) in the ith environment, jth genotype, *k*th replicate within ith environment, $l$th trial, and $m$th incomplete block within the $l$th trial, *k*th replicate at the ith environment, $E_{i }$ is the fixed effect of the *i*th environment, $L_{j}$ is the fixed main effect of the *j*th genotype, $LE_{ij}$ is the random interaction effect between the *i*th environment and the *j*th line assumed to be iid $N\left( {0, \sigma_{gE}^{2} } \right)$, $r_{{k\left( {l,i} \right)}}$ is the random effect of the *k*th replicate within $l$th trial and ith environment assumed to be iid $N\left( {0, \sigma_{r\left( i \right)}^{2} } \right)$, $t_{l\left( i \right)}$ is the random effect of the $l$th trial within ith environment assumed to be iid $N\left( {0, \sigma_{t\left( i \right)}^{2} } \right)$, $b_{{m\left( {l,k,i} \right)}}$ is the random effect of the $m$th incomplete block within the $l$th trial, *k*th replicate at the ith environment assumed iid $N\left( {0, \sigma_{b\left( i \right)}^{2} } \right)$ and $\epsilon_{ijklm}$ assumed $N\left( {0, \sigma_{e\left( i \right)}^{2} } \right)$ represents the random residual plot error associated with the observation $y_{ijklm}$. The variances of replicates, blocks, and error are environment-specific, which is often a realistic assumption (Piepho et al. [14]) and allows a two-stage analysis to be fully equivalent to a single stage analysis (Piepho et al. [14]). Since our data set is very large and we will perform cross-validation, we performed a two-stage analysis which, according to Piepho et al. [14] and Damesa et al. [4], is appropriate if done properly with little difference from the corresponding single-stage analysis. Therefore, in the first stage of the analysis of individual environments, we rewrote the model 1 as

2 $$y_{ijklm} = \mu_{ij} + r_{{k\left( {l,i} \right)}} + t_{l\left( i \right)} + b_{{m\left( {l,k,i} \right)}} + \epsilon_{ijklm} ,$$

where $\mu_{ij} = E_{i} + L_{j} + LE_{ij}$ is the conditional expected value of the *j*th genotype in the ith environment. Here $\mu_{ij}$ was assumed as a fixed effect and defining $\varvec{\mu}_{i} = \left( {\mu_{i1} , \ldots ,\mu_{iJ} } \right)^{T}$, we estimated the best linear unbiased estimates (BLUEs) as $\hat{\varvec{\mu }}_{i} = \left( {\varvec{X}_{i}^{T} {\varvec{\Sigma}}_{i}^{ - 1} \varvec{X}_{i} } \right)^{ - 1} \varvec{X}_{i}^{T} {\varvec{\Sigma}}_{i}^{ - 1} \varvec{y}_{i}$, where $\varvec{X}_{i}$ is a full rank treatment design matrix for $\varvec{\mu}_{i}$ at the ith environment, $\varvec{y}_{i}$ is the plot observations in the ith environment and ${\varvec{\Sigma}}_{i} = {\text{var}}\left( {\varvec{y}_{i} } \right)$ is the non-singular variance–covariance matrix of the plot data in the ith environment, which depends on the experimental design and the variances $\sigma_{r\left( i \right)}^{2}$, $\sigma_{t\left( i \right)}^{2} ,$ $\sigma_{b\left( i \right)}^{2} , \sigma_{e\left( i \right)}^{2}$. We estimated ${\text{var}}(\hat{\varvec{\mu }}_{i} ) = {\varvec{\Psi}}_{i} = \left( {\varvec{X}_{i}^{T} {\varvec{\Sigma}}_{i}^{ - 1} \varvec{X}_{i} } \right)^{ - 1}$ and then with all the information from the first stage, we fitted the second stage model as

3 $$\hat{\mu }_{ij} = E_{i} + L_{j} + LE_{ij} + \gamma_{ij}$$

where $\gamma_{ij}$ is the residual of the *j*th genotype in the ith environment and ${\text{var}}(\varvec{\gamma}_{i} ) = {\varvec{\Psi}}_{i}$ with $\varvec{\gamma}_{i} = \left( {\gamma_{i1} \ldots ,\gamma_{iJ} } \right)^{T}$. Following Smith et al. [17], we decided to fit the second stage model assuming that ${\text{var}}\left( {\gamma_{ij} } \right) = (\omega^{ij} )^{ - 1}$ where $\omega^{ij}$ is the *j*th diagonal element of ${\varvec{\Psi}}_{i}^{ - 1}$, that is, we used weights based on the inverse of the variances of the associated data points (Smith et al. [17]; Welham et al. [22]; Piepho et al. [14]). This approach of using only the diagonal elements of ${\varvec{\Psi}}_{i}^{ - 1}$ is documented by various authors (Smith et al. [17]; Welham et al. [22]; Piepho et al. [14]) and produces almost identical results as when using all the information of ${\varvec{\Psi}}_{i}^{ - 1}$. It is important to point out that in this second stage the term $L_{j}$ that corresponds to the *j*th genotype is assumed now as a random effect identical and independently distributed (iid) $L_{j} \sim N\left( {0,\sigma_{L}^{2} } \right),LE_{ij}$ is exactly as described above, with iid $LE_{ij} \sim N\left( {0,\sigma_{LE}^{2} } \right)$.

Markers can be introduced in the baseline model (3) such that the effect of line ($L_{j}$) can be replaced by $g_{j}$, which is expressed as a linear regression on marker covariates that approximates the genetic value of the *j*th line such that the vector of genetic random effects $\varvec{g} = \left[ {g_{1} , \ldots ,g_{J} } \right]^{T}$ is assumed $\varvec{g}\sim N\left( {{\mathbf{0}},{\mathbf{G}}\sigma_{g}^{2} } \right)$, where $\sigma_{g}^{2}$ is the genetic variance, and **G** is a genomic relationship matrix that is computed using marker data $\varvec{W}$ as $\varvec{G} = \frac{{\varvec{WW'}}}{m}$ [20, 21]. Furthermore, the effect of line ($L_{j}$) can also be replaced by pedigree information $a_{j}$ with the random vector of additive effects $\varvec{a} = \left[ {a_{1} , \ldots ,a_{J} } \right]^{T}$ assumed as $\varvec{a}\sim N\left( {{\mathbf{0}},{\mathbf{A}}\sigma_{a}^{2} } \right)$, where ${\mathbf{A}}$ is the numerical additive relationship matrix derived from pedigree, and $\sigma_{a}^{2}$ is the additive variance.

Similarly for the interaction terms, when genomic information is used, the line × environment interaction $LE_{ij}$ is replaced by $gE_{ij}$ the random effect of the interaction term of the *i*th environment and the *j*th genotype and $\varvec{gE} = \left[ {gE_{11} , \ldots ,gE_{IJ} } \right]^{T} \sim N\left( {{\mathbf{0}},({\mathbf{G}} \otimes \varvec{I}_{I} )\sigma_{gE}^{2} } \right),$ where $\sigma_{gE}^{2}$ is the variance component associated with the genetic × environment interaction. When pedigree is used, $\varvec{aE} = \left[ {aE_{11} , \ldots ,aE_{IJ} } \right]^{T}\sim N\left( {{\mathbf{0}},({\mathbf{A}} \otimes \varvec{I}_{I} )\sigma_{aE}^{2} } \right),$ where $\sigma_{aE}^{2}$ is the variance component of the additive × environment interaction. $\varvec{I}_{I}$ is an identity matrix for environments, and $\otimes$ denotes the Kronecker product.

Therefore, using genomic information, the baseline model becomes

$$\hat{\mu }_{ij} = E_{i} + g_{j} + gE_{ij} + \gamma_{ij}$$

or when using pedigree, the baseline model is defined as

$$\hat{\mu }_{ij} = E_{i} + a_{j} + aE_{ij} + \gamma_{ij}$$

Also, it is important to point out that we obtained the BLUEs with Eq. (2) not only for GY but also for each of the 250 wavelengths (referred to as the spectrometric data $x_{i} \left( {\lambda_{i} } \right), i = 1, \ldots ,250$ mentioned above) with the intention of removing the design effect of each wavelength and using these wavelengths as covariates in the second stage of the analysis in the appropriate way. This process of removing the design effect of each wavelength with Eq. (2) was done on each of the nine different dates on which the wavelengths were measured. This means that BLUEs of each genotype were obtained for GY and for each wavelength in each of the nine time-points under study. We then created a database of $976 \times 3 = 2928$ rows and 2253 columns where the first column contains environments, the second the names of genotypes, the third the BLUEs of GY and the remaining 2250 columns contain the 250 × 9 covariates (wavelengths that are the spectrometric data) resulting from the combinations of the 250 wavelengths and the nine time-points.

### Proposed statistical models including genomic, pedigree, functional regression

Table 1 describes 14 proposed statistical models that will be used on the previously adjusted data for each time-point. Models M1, M2, M3, M4, M9 and M10 are called conventional models, whereas the others (M5, M6, M7, M8, and M11–M14) are newly proposed models that display wavelengths as functional covariates and are called functional regression models. Each of the 14 proposed models was implemented directly using the genotypes, or replacing the genotypes with the pedigree relationship matrix (**A**) or replacing the genotypes with the genomic relationship matrix (**G**). When the 14 models were implemented using the genomic relationship matrix (**G**), we denoted these models as WG; when these models were implemented using the pedigree relationship matrix, we denoted these models as WA; and when these models were implemented using the lines without genomic or pedigree information, the models were denoted as WO.

**Table 1:** Proposed models

Method	Model	Type
M1	$\hat{\mu }_{ij} = E_{i} + g_{j} + \gamma_{ij}$	Conventional
M2	$\hat{\mu }_{ij} = E_{i} + g_{j} + gE_{ij} + \gamma_{ij}$	Conventional
M3	$\hat{\mu }_{ij} = E_{i} + g_{j} + \mathop \sum \limits_{k = 1}^{p} x_{ijk} \beta_{k} + \gamma_{ij}$	Conventional
M4	$\hat{\mu }_{ij} = E_{i} + g_{j} + gE_{ij} + \mathop \sum \limits_{k = 1}^{p} x_{ijk} \beta_{k} + \gamma_{ij}$	Conventional
M5	$\hat{\mu }_{ij} = E_{i} + g_{j} + \mathop \int \limits_{392}^{851} x_{ij} \left( k \right)\beta_{1} \left( k \right)dk + \gamma_{ij}$	Functional Bayesian B-splines
M6	$\hat{\mu }_{ij} = E_{i} + g_{j} + \mathop \int \limits_{392}^{851} x_{ij} \left( k \right)\beta_{1} \left( k \right)dk + \gamma_{ij}$	Functional Bayesian Fourier
M7	$\hat{\mu }_{ij} = E_{i} + g_{j} + gE_{ij} + \mathop \int \limits_{392}^{851} x_{ij} \left( k \right)\beta_{1} \left( k \right)dk + \gamma_{ij}$	Functional Bayesian B-splines basis
M8	$\hat{\mu }_{ij} = E_{i} + g_{j} + gE_{ij} + \mathop \int \limits_{392}^{851} x_{ij} \left( k \right)\beta_{1} \left( k \right)dk + \gamma_{ij}$	Functional Bayesian Fourier basis
M9	$\hat{\mu }_{ij} = E_{i} + g_{j} + \mathop \sum \limits_{k = 1}^{p} x_{ijk} \beta_{k} + \mathop \sum \limits_{k = 1}^{p} x_{ijk} \beta_{ki} + \gamma_{ij}$	Conventional
M10	$\hat{\mu }_{ij} = E_{i} + g_{j} + gE_{ij} + \mathop \sum \limits_{k = 1}^{p} x_{ijk} \beta_{k} + \mathop \sum \limits_{k = 1}^{p} x_{ijk} \beta_{ki} + \gamma_{ij}$	Conventional
M11	$\hat{\mu }_{ij} = E_{i} + g_{j} + \mathop \int \limits_{392}^{851} x_{ij} \left( k \right)\beta_{1} \left( k \right)dk + \mathop \int \limits_{392}^{851} x_{ij} \left( k \right)\beta_{2i} \left( k \right)dk + \gamma_{ij}$	Functional Bayesian B-splines basis
M12	$\hat{\mu }_{ij} = E_{i} + g_{j} + \mathop \int \limits_{392}^{851} x_{ij} \left( k \right)\beta_{1} \left( k \right)dk + \mathop \int \limits_{392}^{851} x_{ij} \left( k \right)\beta_{2i} \left( k \right)dk + \gamma_{ij}$	Functional Bayesian Fourier basis
M13	$\hat{\mu }_{ij} = E_{i} + g_{j} + gE_{ij} + \mathop \int \limits_{392}^{851} x_{ij} \left( k \right)\beta_{1} \left( k \right)dk + \mathop \int \limits_{392}^{851} x_{ij} \left( k \right)\beta_{2i} \left( k \right)dk + \gamma_{ij}$	Functional Bayesian B-splines basis
M14	$\hat{\mu }_{ij} = E_{i} + g_{j} + gE_{ij} + \mathop \int \limits_{392}^{851} x_{ij} \left( k \right)\beta_{1} \left( k \right)dk + \mathop \int \limits_{392}^{851} x_{ij} \left( k \right)\beta_{2i} \left( k \right)dk + \gamma_{ij}$	Functional Bayesian Fourier basis

In the 14 proposed models given in Table 1, when pedigree is used instead of markers, the random genetic $g_{j}$ term is replaced by the random additive effect $a_{j}$. While the interaction term $gE_{ij}$ is replaced by the random interaction term $aE_{ij}$. In models M3, M4, M9 and M10 $x_{ijk}$ represent the $k$th discretized spectrometric data measured on the $j$th genotype in the $i$th environment with $k = 1,2, \ldots ,250$ and we need to remember that $x_{ijk}$ are predicted means obtained in the first stage analysis. $\beta_{k}$ is the beta regression coefficient for the $k$th band that will be estimated. The functional regression models M5 and M6 add the 250 wavelengths to M1 as a functional covariate constructed over the interval between 392 and 851 nm, which are the minimum and maximum values at which the 250 wavelength bands of the reflectance data were measured. Therefore, $x_{ij} \left( k \right)$ is the functional predictor and represents the value of a continuous underlying process evaluated at wavelength $k$, $\beta_{1} \left( k \right)$ is the functional regression beta coefficient for the functional part of models M5, M6, M7, M8, M11, M12, M3 and M14, which is a function of the wavelength $k$. In this context, the integral of the product replaces the sum of products $(\mathop \sum \nolimits_{k = 1}^{p} x_{ijk} \beta_{k} )$ in the conventional linear regression model given in M3. Models M5, M7, M11, M13 should be called Bayesian B-splines since they will be implemented under a Bayesian approach using the B-splines as basis expansion and models M6, M8, M12, M14 will be called the Bayesian Fourier models since they use the Fourier basis. Model M9 adds to M3 the band by environment (B × E) interaction between the *i*th environment and the *k*th band and $\beta_{ki}$ represents the beta regression coefficient corresponding to the *k*th band measured in the *i*th environment. Model M11 adds to M5 and Model M12 adds to M6 the interaction between environment and the functional regression predictor that represents the reflectance data and $\beta_{2i} \left( k \right)$ is the coefficient function corresponding to the functional part that represents the interaction between the *i*th environment and the *k*th band. Model M13 adds to M7 and model M14 adds to M8 the interaction between environment and the functional regression predictor.

The proposed functional regression models M5, M6, M7, M8, M11, M12, M13, and M14 are among the most popular functional regression models, where the responses are scalars and some of the covariates are functions. For this reason, the response variable $(\hat{\mu }_{ij} )$ is scalar in all the proposed models and represents grain yield (GY). Apart from the general subdivision of the 14 models in conventional versus functional regression, it is useful to point out that the 14 models also differ in various other aspects based on their conventional and functional regression components, for example, models that do not include the $gE_{ij}$ interaction term (M1, M3, M5, M6, M9, M11, and M12) versus models that do include the $gE_{ij}$ interaction term (M2, M4, M7, M8, M10, M13, and M14). Regarding the functional regression models, models M5, M7, M11, and M13 had the B-spline basis, whereas models M6, M8, M12, and M4 had the Fourier basis. Models M9–M14 include the B × E interaction between the *i*th environment and the *k*th band, but models M9 and M10 assessed this interaction using the conventional approach, whereas models M11–M14 fitted the B × E interaction by means of the functional regression model. Additional details about functional regression models can be found in Ramsay and Silverman [15].

#### Preprocessing the functional regression models (M5–M8 and M11–M14)

For the estimation of the parameters of the functional regression models M5, M6, M7, M8, M11, M12, M13, and M14, first we need to know the exact form of the functional covariate [$x\left( k \right)$], but this only was observed in discrete points. A traditional approach is to assume that the functional covariate ($x\left( k \right)$) and the functional regression beta coefficients ($\beta_{1} \left( k \right)$) can be represented by the linear combination of a truncated basis. With this the high dimensional problem is reduced to standard linear model, as we will describe next. First, we represent covariable curves as

4 $$x_{ij} \left( k \right) = \mathop \sum \limits_{l = 1}^{L} c_{ijl} \phi_{l} \left( k \right)$$

where $\phi_{1} \left( k \right), \ldots ,\phi_{L} \left( k \right)$ is a truncate basis (B-splines, Fourier basis) and $c_{ijl}$ is the coefficient corresponding to the $ij{\text{th}}$ individual (environment-line combination) of the function $\phi_{l} (k$). Assuming that each curve was observed in $\varvec{k} = [k_{1} , \ldots , k_{m} ]^{T}$, then in vector form

$$x_{ij} \left( \varvec{k} \right) = \left[ {\begin{array}{*{20}c} {\mathop \sum \limits_{l = 1}^{L} c_{ijl} \phi_{l} \left( {k_{1} } \right)} \\ \vdots \\ {\mathop \sum \limits_{l = 1}^{L} c_{ijl} \phi_{l} \left( {k_{m} } \right)} \\ \end{array} } \right] = \left[ {\begin{array}{*{20}c} {\phi_{1} \left( {k_{1} } \right)} & \cdots & {\phi_{L} \left( {k_{1} } \right)} \\ \vdots & \ddots & \vdots \\ {\phi_{1} \left( {k_{m} } \right)} & \cdots & {\phi_{L} \left( {k_{m} } \right)} \\ \end{array} } \right]\varvec{c}_{ij} = {\varvec{\Phi}}\varvec{c}_{ij}$$

where $\varvec{c}_{ij}^{T} = \left[ {c_{ij1} , \ldots ,c_{ijL} } \right]$. Therefore, the values of $\varvec{c}_{ij}$ that best represent $x_{ij} \left( \varvec{k} \right)$ in terms of minimizing $\left[ {x_{ij} \left( \varvec{k} \right) - {\varvec{\Phi}}\varvec{c}_{ij} } \right]^{T} \left[ {x_{ij} \left( \varvec{k} \right) - {\varvec{\Phi}}\varvec{c}_{ij} } \right]$ are given by

5 $$\hat{\varvec{c}}_{ij} = \left[ {{\varvec{\Phi}}^{T} {\varvec{\Phi}}} \right]^{ - 1} {\varvec{\Phi}}^{T} x_{ij} \left( \varvec{k} \right)$$

If $\beta_{1} \left( k \right) = \mathop \sum \nolimits_{s = 1}^{S} d_{s} \psi_{s} \left( k \right)$ is the representation of $\beta_{1} \left( k \right)$ in terms of another truncated basis, $\psi_{1} \left( k \right), \ldots ,\psi_{S} \left( k \right),$ the model M5 can be rewritten as

6 $$\begin{aligned} \hat{\mu }_{ij} & = E_{i} + g_{j} + \mathop \sum \limits_{s = 1}^{S} d_{s} \int x_{ij} \left( k \right)\psi_{s} \left( k \right)dk + \gamma_{ij} \\& = E_{i} + g_{j} + \mathop \sum \limits_{s = 1}^{S} d_{s} x_{ijs} + \gamma_{ij} \\ & = E_{i} + g_{j} + \varvec{x}_{ij}^{T} \varvec{d} + \gamma_{ij} , \\ \end{aligned}$$

where $x_{ijs} = \int x_{ij} \left( k \right)\psi_{s} \left( k \right)dk$, $\varvec{x}_{ij}^{T} = \left[ {x_{ij1} , \ldots ,x_{ijS} } \right],$ and $\varvec{d}^{T} = \left[ {d_{1} , \ldots ,d_{S} } \right]$ is an unknown vector of coefficients related to the effect of the functional covariate. The elements of $\varvec{x}_{ij}^{T}$ can be obtained from the covariate representation given previously

7 $$x_{ij} \left( k \right) = \mathop \sum \limits_{l = 1}^{L} \hat{c}_{ijl} \phi_{l} \left( k \right)$$

where $\hat{c}_{ijl}$, $l = 1, \ldots ,{\text{L}}$, are the elements of $\hat{\varvec{c}}_{ij}$ obtained in Eq. (5). Substituting (7) in $x_{ijs} = \int x_{ij} \left( k \right)\psi_{s} \left( k \right)dk$, the elements of $\varvec{x}_{ij}^{T}$ explicitly can be computed as

$$x_{ijs} = \int x_{ij} \left( k \right)\psi_{s} \left( k \right)dk = \mathop \sum \limits_{l = 1}^{L} \hat{c}_{ijl} \int \phi_{l} \left( k \right)\psi_{s} \left( k \right)dk,$$

where the coefficients $\hat{c}_{ijl}$ are given in Eq. (5). Then by making $J_{ls} = \int \phi_{l} \left( k \right)\psi_{s} \left( k \right)dk$, we have that the required $\varvec{x}_{ij}$ can be approximated as

8 $$\varvec{x}_{ij} = \left[ {\begin{array}{*{20}c} {\mathop \sum \limits_{l = 1}^{L} \hat{c}_{ijl} J_{l1} } \\ \vdots \\ {\mathop \sum \limits_{l = 1}^{L} \hat{c}_{ijl} J_{lS} } \\ \end{array} } \right] = \left[ {\begin{array}{*{20}c} {\hat{c}_{ij1} J_{11} } & \cdots & {\hat{c}_{ijL} J_{L1} } \\ \vdots & \ddots & \vdots \\ {\hat{c}_{ij1} J_{1S} } & \cdots & {\hat{c}_{ijL} J_{LS} } \\ \end{array} } \right] = \left[ {\begin{array}{*{20}c} {\varvec{J}_{1}^{T} } \\ \vdots \\ {\varvec{J}_{S}^{T} } \\ \end{array} } \right]\hat{\varvec{c}}_{ij} = \varvec{ J\hat{c}}_{ij}$$

where $\varvec{J}_{s}^{T} = \left[ {J_{1s} , \ldots ,J_{Ls} } \right]$ and $\hat{\varvec{c}}_{ij}^{T} = \left[ {\hat{c}_{ij1} , \ldots ,\hat{c}_{ijL} } \right]$. Therefore, since we obtained $\varvec{x}_{ij}$, we can implement M5 given in Eq. (6) using conventional Bayesian or classical modeling. See Ramsay and Silverman [15] for more details and considerations.

It is important to point out that the same logic was used for the rest of the models that have a functional component (M6, M7, M8, M11, M12, M13, M14) in order to obtain their corresponding $\varvec{x}_{ij}$ components. However, calculating $\varvec{x}_{ij}^{T}$ using Eq. (8) can seem somewhat complex to those not familiar with functional regression or with matrices; for this reason, “Appendix 1” gives the implementation of each of the 14 proposed models. Also, for models that include a functional component (M5, M6, M7, M8, M11, M12, M13, M14), the corresponding code for building the $\varvec{x}_{ij}^{T}$ component using $S = L = 21$ basis expansion is provided. Implementation of the proposed models was carried out under the Bayesian paradigm; for this reason, in the next section we provide information about the prior distributions we used.

#### Assumptions on priors

For the beta coefficient of the *i*th environment, we assumed a $N\left( {0,10000} \right),$ for $\sigma_{g}^{2}$, a scaled inverse Chi square distribution $\chi^{ - 2} (\sigma_{g}^{2} |S_{g} , df_{g}$) with scale factor $S_{g}$ and degrees of freedom $df_{g} > 0$; and for $\sigma_{e}^{2} ,$ also a scaled inverse Chi square distribution $\chi^{ - 2} (\sigma_{e}^{2} |S_{e} , df_{e}$) with scale factor $S_{e} = 2E10$ and degrees of freedom $df_{e} = 2E10$. This scale and degrees of freedom of the variance component of the error were choosen in this way to be able to implement the proposed two stage analysis in the BGLR package because these values warranty a prior distribution highly concentrated about 1, with very small variability. For $\sigma_{gE}^{2}$, we assumed a scaled inverse Chi square distribution $\chi^{ - 2} (\sigma_{gE}^{2} |S_{gE} , df_{gE}$) with scale factor $S_{gE}$ and degrees of freedom $df_{gE} > 0$.

For the beta coefficients of each of the bands ($$\beta_{k} , k = 1, \ldots ,p),$$ we used $$N\left( {0,\sigma_{{\beta_{1} }}^{2} } \right)$$ for $$\sigma_{{\beta_{1} }}^{2} \sim \chi^{ - 2} (\sigma_{{\beta_{1} }}^{2} |S_{{\beta_{1} }} , df_{{\beta_{1} }})$$. For the beta coefficients of each component of the interaction terms between environments and bands $$(\beta_{ik}, i = 1, \ldots ,3;k = 1, \ldots ,p)$$, we used $$N\left( {0,\sigma_{{\beta_{2} }}^{2} } \right)$$ for $$\sigma_{{\beta_{2} }}^{2} \sim \chi^{ - 2} (\sigma_{{\beta_{2} }}^{2} |S_{{\beta_{2} }} , df_{{\beta_{2} }})$$. To control the smoothness of the parameter functions, we use a multivariate normal distribution as prior distribution for $$\varvec{d}$$ with mean the vector **0** and covariance matrix $$\sigma_{d}^{2} \varvec{P}^{ - 1} ,$$ where $$\varvec{P} = \left\{ {P_{ij} } \right\}$$ is a penalty matrix, $$P_{ij} = \int \nolimits_{a}^{b} \psi^{\prime \prime }_{i} \left( t \right)\psi^{\prime \prime }_{j} \left( t \right)dt$$, $$i, j = 1, \ldots ,{\text{S}},\,\psi_{i} ''\left( t \right)$$ is the second derivate of the $$\psi_{i} \left( t \right)$$, and $$1/\sigma_{d}^{2}$$ is a smoothing parameter, and the prior for $$\sigma_{d}^{2}$$ was $$\chi^{ - 2} (\sigma_{d}^{2} |S_{d} , df_{d}$$). In a similar way, for the beta coefficients of the basis ($$\varvec{d}_{2}$$) corresponding to the interaction terms between environments and the functional covariates ($$\beta_{2i} \left( \cdot \right)$$), we assume $$\varvec{d}_{2} \sim N\left( {0,\sigma_{dI}^{2} \varvec{P}_{d}^{ - 1} } \right),$$ where $$\varvec{P}_{d} = \left\{ {P_{ij}^{\varvec{*}} } \right\}$$ is a penalty matrix, $$P_{ij}^{*} = \int \nolimits_{a}^{b} \psi_{i} ''\left( t \right)\psi_{j} ''\left( t \right)dt,\,\,i, j = 1, \ldots ,S_{dI} ,\,\psi_{i} ''\left( t \right)$$ is the second derivate of the $$\psi_{i} \left( t \right)$$, and $$1/\sigma_{dI}^{2}$$ is a smoothing parameter and for $$\sigma_{dI}^{2} \sim\chi^{ - 2} (\sigma_{dI}^{2} |S_{dI} , df_{dI}$$). Here we are representing $$\beta_{2i} \left( t \right)$$ as $$\mathop \sum \nolimits_{i = 1}^{{S_{dI} }} d_{Ii} \psi_{i} \left( t \right)$$. When used the Fourier basis we set $$P_{11}^{\varvec{*}} = P_{11} = 1$$, to avoid a degenerate prior distribution concentrated in 0 for the the first elements of $$\varvec{d}$$ and $$\varvec{d}^{\varvec{*}} .$$ For using the BGLR package, the corresponding desing matrix is post-multiplicaed by the square root matrix of $$\varvec{P}^{ - 1}$$ ($$\varvec{P}_{d}^{ - 1} )$$, $$\varvec{P}^{ - 1/2}$$ ($$\varvec{P}_{d}^{ - 1/2} )$$. All the priors used were weakly informative with the exception of the variance component of error **(** $$\sigma_{e}^{2} )$$ which was totally informative and concentrated at 1 in order to be able to correctly implement the two stage analysis. Note that when the number of basis ($$L)$$ used is small (less than 30), $$P_{d}^{ - 1}$$ can be assumed an identity matrix.

The 14 proposed models were implemented in the BGLR R-package (de los Campos and Pérez-Rodríguez [5]) using the hyper-parameters as set, using the rules of this package with 30,000 iterations and a burn-in period of 20,000. First, models were fitted to the entire data set to evaluate goodness-of-fit to the training data; they were then implemented through the cross-validation described in the next section.

#### Assessing the models’ prediction accuracy

We used two schemes for assessing the prediction accuracy of the 14 models; one consists of ten training (trn)–testing (tst) random partitions with 50% of the lines assigned to the training data set and the remaining 50% to the testing data set. The other scheme is also a ten trn–tst random partition, but with 10% of the lines in one environment assigned to training and 90% to testing; under this scenario, the two environments maintained the complete number of lines.

The first cross-validation scheme was used to examine the prediction accuracies of the 14 proposed models given in the previous section; for each random partition in each environment, we used 488 (50%) lines for training and 488 (50%) for testing (50CV). This means that from the whole data set comprising the three environments, the training data set that we used consisted of 1464 observations (50%) and the validation data set we used, the remaining 1464 observations (50%). This type of cross-validation mimics a situation where the researcher wants to predict 50% of the lines in some environments; however, the lines whose phenotypes we wanted to predict were measured in at least one of the environments (that is, they were not missing in all the environments).

The second cross-validation scheme was only used for evaluating models M13 and M14 and consisted of removing 90% of the lines in one environment and predicting them using all the lines in the other two environments (90CV). This cross-validation (leaving 90% of the lines unobserved in one environment) mimics the situation where all the information of the lines is available in all environments except one, where only 10% of the lines have phenotypic data.

For both random cross-validation schemes, we used the Pearson correlation between the predicted values of the model and the observed BLUP value for GY as a measure of prediction accuracy calculated for each environment. We reported the average and the standard error (SE) of the 10 Pearson correlations resulting from the ten trn–tst random partitions implemented. It is also important to point out that we used the same split (of the ten trn–tst random partitions) in the 14 models to ensure fair comparisons.

## Results

In this section, we present the main results of the implementation of the proposed models. The results are given in seven sections. The first section provides a descriptive summary of how similar the environments, time points and bands are. The second section compares the proposed models with genomic data (WG), with pedigree information (WA) and without marker or pedigree data (WO). The third section compares the models in each environment for the 9 time-points. The fourth section compares the prediction accuracies of the proposed models between environments at each of the 9 time-points. The fifth section compares the 9 time-points in each environment for models M5, M7, M11, and M13. The sixth section compares the computing time needed to implement each of the proposed models; the random cross-validation shown in these four sections is the 50CV. Finally, the seventh section presents the prediction accuracy of models M11 and M13 for the 9 time-points and for each environment when in one environment, 90% of the lines were missing and are predicted using all the data in the other two environments; the random cross-validation in this section is the 90CV.

### Descriptive summary of how similar the environments, time points and bands are

We found that the genetic correlation in yield between the Drought and Irrigated environments was negative and low (−0.1418), while the correlation between the Drought and Reduced Irrigated environments was positive and moderately high (0.508) and the correlation between the Irrigated and the Reduced Irrigated environments was also negative and low (−0.034). On the other hand, Table 2 provides the correlations between the 9 time-points (for each time-point we used as response, the average of the 250 bands); in the Drought environment, only 7 correlations out of 36 were larger than 0.4, in the Irrigated environment, we found 10 out of 36 correlations were larger than 0.4, while in the Reduced Irrigated environment, we found 11 out of 36 correlations were larger than 0.4. Note that the the highest correlation is not with the nearest neighbor time point.

**Table 2:** Pearson correlations of the time-points for each environment

Time	1	2	3	4	5	6	7	8	9
Drought									
1	1.000	0.754	0.257	0.319	−0.066	−0.069	0.213	−0.020	−0.065
2	*0.754*	1.000	0.201	0.504	0.096	0.248	−0.010	0.364	0.031
3	0.257	0.201	1.000	0.341	0.256	0.185	0.216	−0.118	0.138
4	0.319	*0.504*	0.341	1.000	0.505	0.349	0.205	0.420	0.160
5	−0.066	0.096	0.256	*0.505*	1.000	0.323	−0.009	0.596	0.063
6	−0.069	0.248	0.185	0.349	0.323	1.000	−0.277	0.631	0.221
7	0.213	−0.010	0.216	0.205	−0.009	−0.277	1.000	−0.401	0.489
8	−0.020	0.364	−0.118	*0.420*	*0.596*	*0.631*	*−0.401*	1.000	0.090
9	−0.065	0.031	0.138	0.160	0.063	0.221	*0.489*	0.090	1.000
Irrigated									
1	1.000	0.309	0.659	0.474	0.401	−0.097	−0.099	0.065	−0.031
2	0.309	1.000	0.168	0.406	−0.416	0.832	0.062	0.876	0.008
3	*0.659*	0.168	1.000	0.743	0.460	−0.115	0.123	0.062	0.179
4	*0.474*	*0.406*	*0.743*	1.000	0.298	0.208	0.140	0.428	0.097
5	*0.401*	*−0.416*	*0.460*	0.298	1.000	−0.491	−0.072	−0.329	−0.003
6	−0.097	*0.832*	−0.115	0.208	−*0.491*	1.000	0.137	0.908	0.039
7	−0.099	0.062	0.123	0.140	−0.072	0.137	1.000	0.084	0.882
8	0.065	0.876	0.062	*0.428*	−0.329	*0.908*	0.084	1.000	0.002
9	−0.031	0.008	0.179	0.097	−0.003	0.039	*0.882*	0.002	1.000
Reduced Irrigated									
1	1.000	0.623	0.741	0.531	0.465	−0.007	−0.190	0.012	−0.053
2	*0.623*	1.000	0.485	0.538	0.252	0.248	−0.136	0.183	−0.049
3	*0.741*	*0.485*	1.000	0.640	0.434	0.090	−0.102	0.149	0.025
4	*0.531*	*0.538*	*0.640*	1.000	0.600	0.044	0.023	0.081	0.117
5	*0.465*	0.252	*0.434*	*0.600*	1.000	−0.263	−0.109	−0.426	0.037
6	−0.007	0.248	0.090	0.044	−0.263	1.000	0.038	0.804	0.128
7	−0.190	−0.136	−0.102	0.023	−0.109	0.038	1.000	0.080	0.841
8	0.012	0.183	0.149	0.081	*−0.426*	*0.804*	0.080	1.000	0.050
9	−0.053	−0.049	0.025	0.117	0.037	0.128	*0.841*	0.050	1.000

Italic values indicate the Pearson correlation larger than 0.4 for each time point

In Figs. 8, 9 and 10, we can see that most of the bands are highly correlated with the correlation between bands being stronger in the Irrigated and Reduced Irrigated environment and a weaker in the Drought environment. Also, it is very important to point out that the patterns of similarity in the correlations are very similar between the Irrigated and Reduced Irrigated environments. On the other hand, since the bands are very highly correlated, one can opt to omit some bands from the analysis without significant loss of information. This option was taken into account by Montesinos-López et al. [13] (reducing the dimension of the bands using principal component analysis and working only some bands that showed high heritability) but although the results were a little faster in terms of computational implementation, no gain was observed in terms of predicción accuracy. For this reason, in this paper we used all the available bands and we reduced the dimension of the bands by functional data analysis.

### Comparing the models with genomic (WG), pedigree (WA) information and without genomic and without pedigree (WO) information

**Fig. 1:**
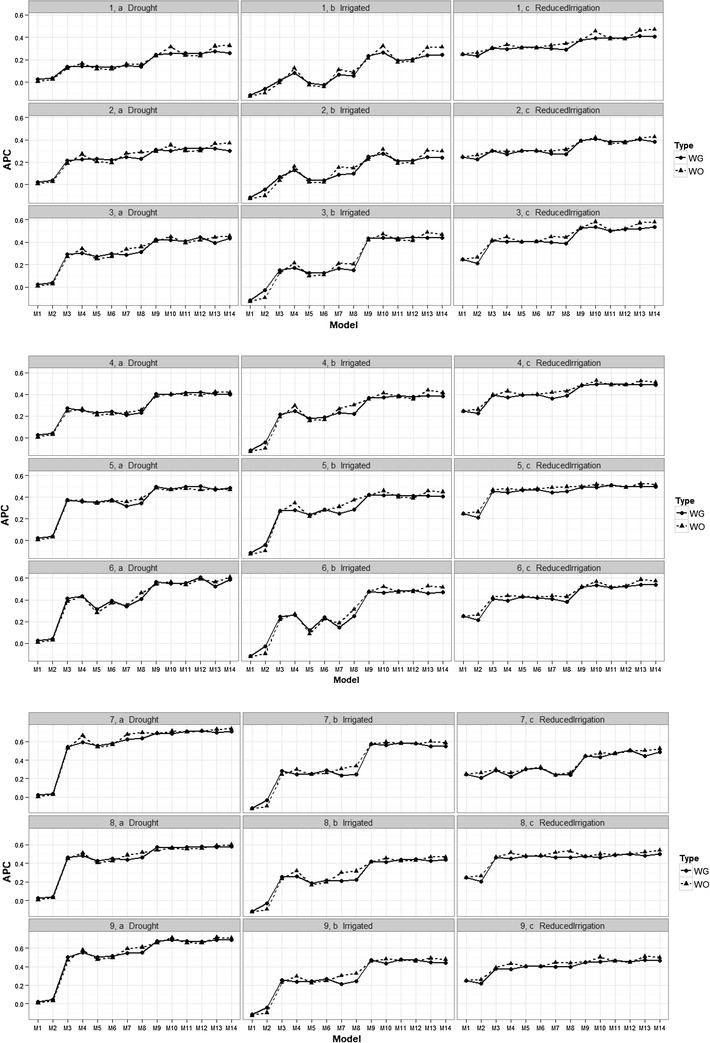
Prediction accuracy of the proposed models for the time-environment combination, with the genomic relationship matrix (WG) and without the genomic relationship matrix (WO). The reported prediction accuracy resulted from the average of the ten trn–tst random partitions of the Pearson correlation between observed and predicted values (APC) (50CV random cross-validation)

**Fig. 2:**
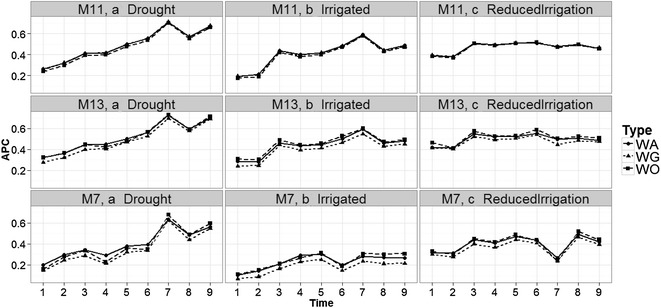
Prediction accuracy for each time-point in the three environments and models M7, M11 and M13 with the genomic relationship matrix (WG), with the pedigree relationship matrix (WA) and without the genomic (and pedigree) relationship matrix (WO). The reported prediction accuracy resulted from the average of the ten trn–tst random partitions of the Pearson correlation between observed and predicted values (APC) (50CV random cross-validation)

Results show that there were no differences in the prediction accuracy of the proposed models when the genomic relationship matrix was taken into account (WG) compared to when the genomic relationship matrix was ignored (WO) (Fig. 1). Some models without genomic information produced predictions that were a little better, for example, at time-point 2: in the Drought environment, the predictions are a little better for models M4, M7, M8, M10, M13 and M14, in the Irrigated environment they are a little better for models M4, M7, M8, M13, and M14, and in the Reduced Irrigated environment, the predictions are better for models M2, M8 and M14. At time-point 4, the predictions in the Irrigated environment are a little better for models M4, M7, M8, M10, M13 and M14, while in the Reduced Irrigated environment, the predictions are a little better for models M4, M7, M8, M10 and M13. At time-point 6, in the Drought environment, the predictions in favor of the models without genomic information are given in model M8; in the Irrigated environment, the models with a little better predictions are M8, M10, M13 and M14, while in the Reduced Irrigation environment, the models without genomic information were slightly better in all models except in models M1, M3, M5, M6, M7, M8, M9, M10, M11 and M12. Also in Fig. 2 we can see that there are no relevant differences in terms of prediction accuracy using genomic information, pedigree information and without pedigree and genomic information. However, in general, using pedigree information produced predictions that were a little better than when using the genomic information, or ignoring both the genomic and pedigree information. Also in Fig. 2, we can see that in general, the later the time-point, the better the predictions; however, this trend is clearer in the Drought environment. In the Drought and Irrigated environments, the best predictions were observed at time-point 7, but in general, at time-point 6, the predictions are comparable to those of the latter points. It is important to point out that all the standard errors (SE) of each APC resulting of all the proposed models are given in “Appendix 3”.

### Comparing models in each environment for each time-point

**Fig. 3:**
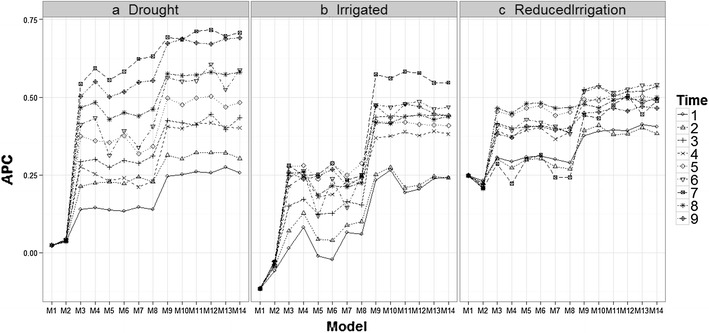
Prediction accuracy of the proposed models in the three environments for the 9 time-points versus the average of the ten trn–tst random partitions of the Pearson correlation between observed and predicted values (APC) (50CV random cross-validation)

**Fig. 4:**
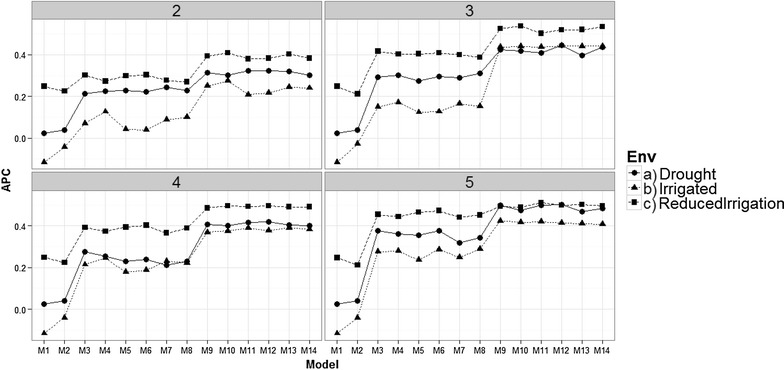
Comparison of prediction accuracy between environments of the proposed models for time-points 2–5 versus the average of the ten trn–tst random partitions of the Pearson correlation between observed and predicted values (APC) (50CV random cross-validation)

**Fig. 5:**
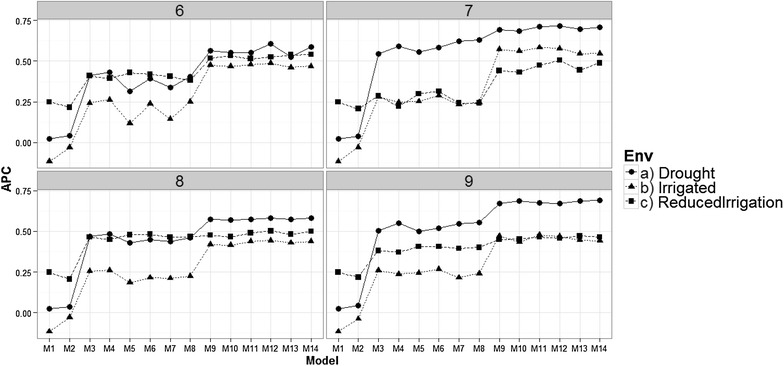
Comparison of the prediction accuracy between environments of the proposed models for time-points 6–9 versus the average of the ten trn–tst random partitions of the Pearson correlation between observed and predicted values (APC) (50CV random cross-validation)

**Fig. 6:**
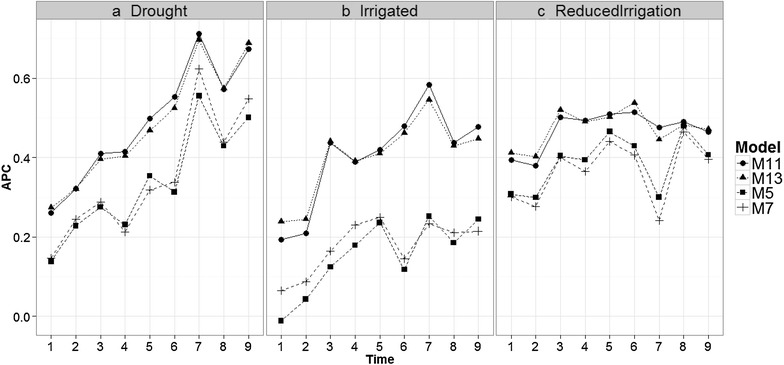
Comparison of time-points (1–9) versus the average of the ten trn–tst random partitions of the Pearson correlation between observed and predicted values (APC) (50CV random cross-validation) in the Drought, Irrigated and Reduced Irrigation environments for models M5, M7, M11, M13

All results given in Figs. 3, 4, 5, and 6 were obtained taking into account the genomic relationship matrix (WG), but similar results were observed with the pedigree relationship matrix (WA) and without pedigree and genomic data (WO). Figure 3 shows that there are differences in prediction accuracy among the 14 proposed models. It is clear that the worst models in terms of prediction accuracy were models M1 and M2 (without information of bands) and the best models were M9–M14. However, there are no strong differences in terms of prediction accuracies between environments, but it is interesting to point out that in the three environments, the worst predictions were observed at time-points 1 and 2 and the second worst were observed at time-point 4. The best predictions were observed at time-point 9 for models M13 to M14 in the Drought environment, and for models M9, M11 and M12 in the Irrigated environment. However, in the Reduced Irrigation environment, the best predictions for models M13 to M14 were observed at time-point 6. In the Reduced Irrigated (Fig. 3) environment, we observed few differences in prediction accuracy between the 9 time-points, while in the Drought and Irrigated environments, we observed larger differences between time-points in terms of prediction accuracy.

### Comparison between environments for each of the 9 time-points

Figures 4 and 5 show that there are differences in prediction accuracies between environments at time-points 1–9. At time-point 2, 3, 4 and 5, the best predictions are in the Reduced Irrigation environment, and at time-points 7 and 9, the best predictions are in the Drought environment, while at the remaining time-points (6 and 8), the predictions in the three environments are more similar. However, it is important to point out that the prediction accuracies for time-points 2, 3, 4 and 5 are around 0.2–0.5 for the Drought and Reduced Irrigated environments. On the other hand, most of the predictions for time-points 6, 7, 8 and 9 are higher than 0.35. Furthermore, at time-points 7 and 9, the worst predictions were observed in the Reduced Irrigation environment and the second worst in the Irrigated environment.

### Comparing time-points in each environment for models M5, M7, M11, and M13

Figure 6 shows that the prediction accuracies of models M5, M7, M11 and M13 are different; in general, the earlier the time point, the worse the predictions. This is expected because in two environments (Drought and Irrigated), the worst predictions were observed at time-points 1 and 2. At each time-point, the prediction accuracies of models M5 and M7 are similar and consistently lower than the prediction accuracies of models M11 and M13 (which gave similar predictions).

It is important to note that the main difference between M5 and M7 is the interaction term ($$gE_{ij} )$$, which is ignored in M5 but present in M7. Since these two models have similar prediction accuracies, this result indicates that the interaction term did not help increase prediction accuracy. The same argument applies for M11 and M13. Model M5 has the main effects of environments and lines as a predictor plus the information on the bands as functional covariates, whereas model M11 takes into account the interaction between environments and bands (functional covariate) in addition to these terms. Therefore, the differences between M5 and M11 can be attributed to the interaction between environments and bands as a functional covariate. Also, since the difference between M7 and M11 is the interaction between environments and bands, the differences in prediction accuracy can also be attributed to this interaction.

The differences between models M5, M7, M11 and M13 were larger in the Reduced Irrigation environment (Fig. 6) at time-point 7. Also, it is important to point out that in the Drought environment, the best predictions belong to time-points 7 and 9 for the four models (M5, M7, M11 and M13), while in the Irrigated environment, the best predictions were observed at time-points 7 only for models M11 and M13. In the Reduced Irrigation environment, the best predictions for the two models (M11 and M13) were observed at time-point 6. Similar behavior was observed at all time-points for the other models (see Figs. 11 and 12 in “Appendix 2”).

### Comparing the models’ computational speed for implementation

**Fig. 7:**
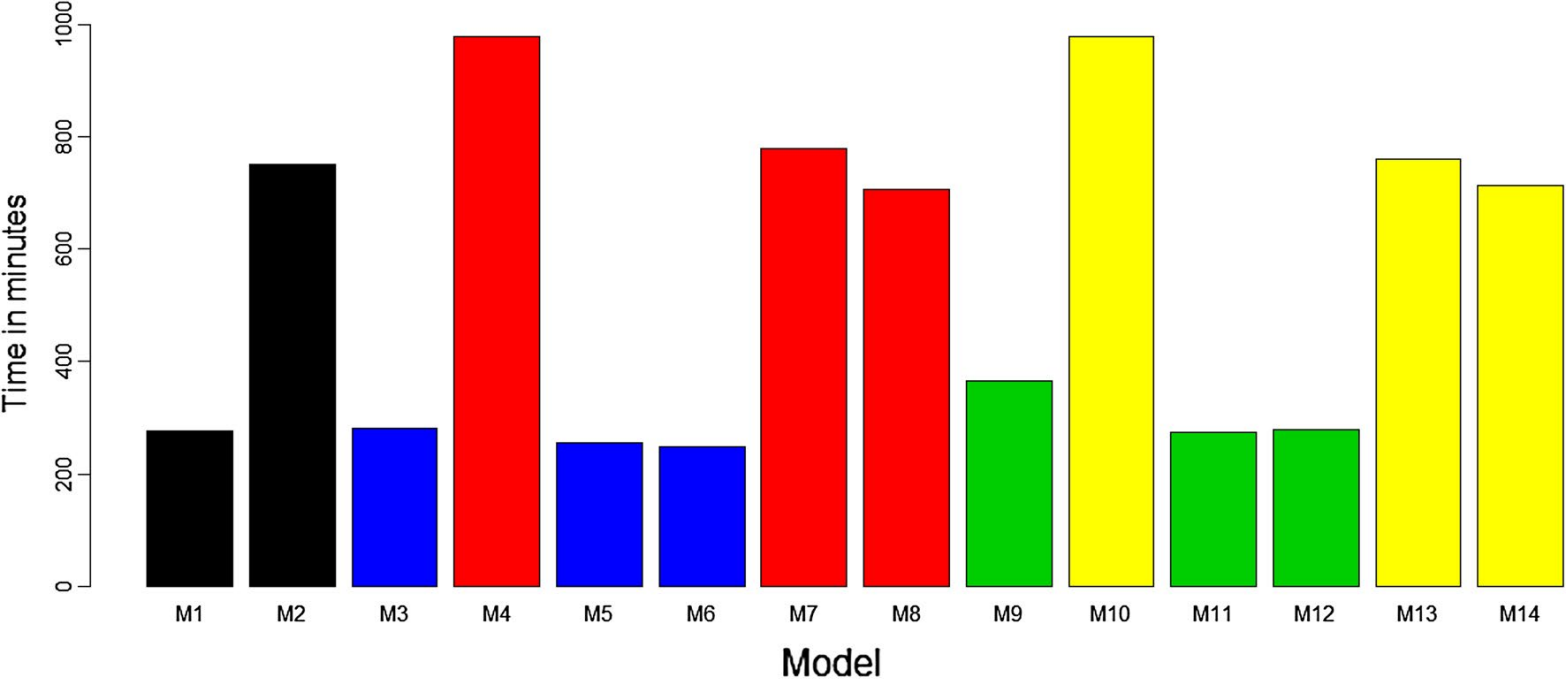
Computational speed (in minutes) required for implementing each proposed model

**Fig. 8:**
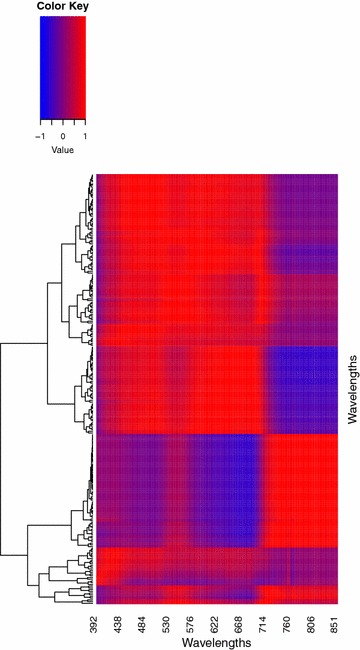
Heatmap for the 250 bands in environment Drought. In the x-axis the bands are presented from the lowest to largest wavelength measured (392–851 nm), while in the y-axis the wavelengths are clustered

**Fig. 9:**
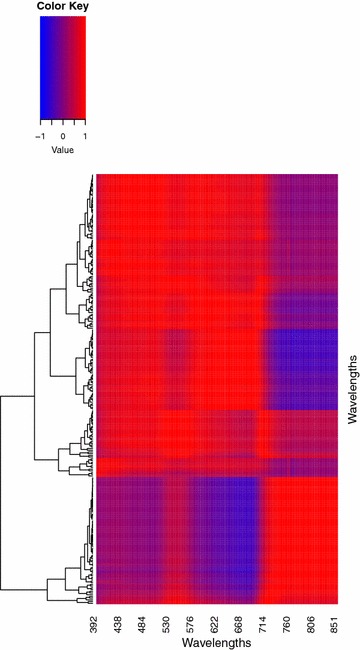
Heatmap for the 250 bands in environment Irrigated. In the x-axis the bands are presented from the lowest to largest wavelength measured (392–851 nm), while in the y-axis the wavelengths are clustered

**Fig. 10:**
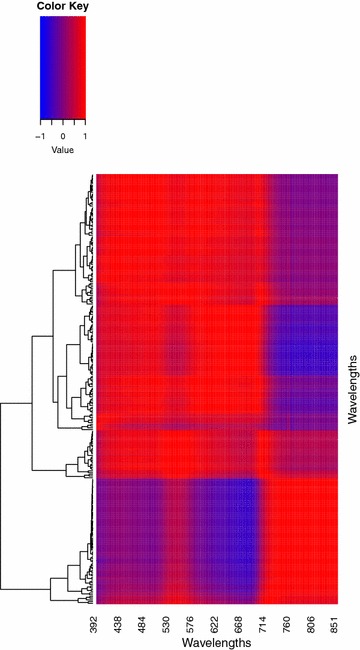
Heatmap for the 250 bands in environment Reduced Irrigated. In the x-axis the bands are presented from the lowest to largest wavelength measured (392–851 nm), while in the y-axis the wavelengths are clustered

**Fig. 11:**
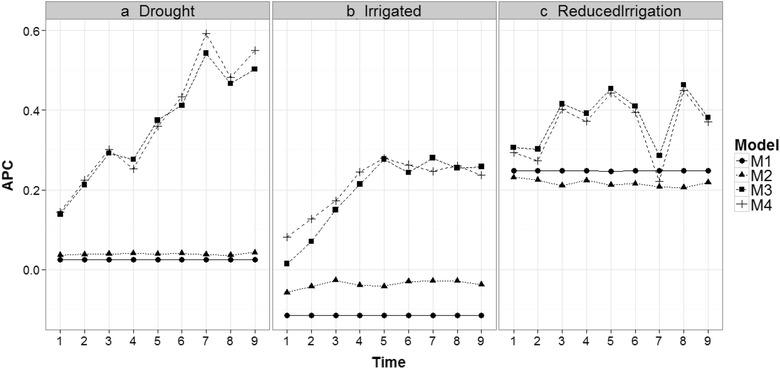
Comparison of time-points in the Drought, Irrigated and Reduced Irrigation environments for models M1, M2, M3, and M4

**Fig. 12:**
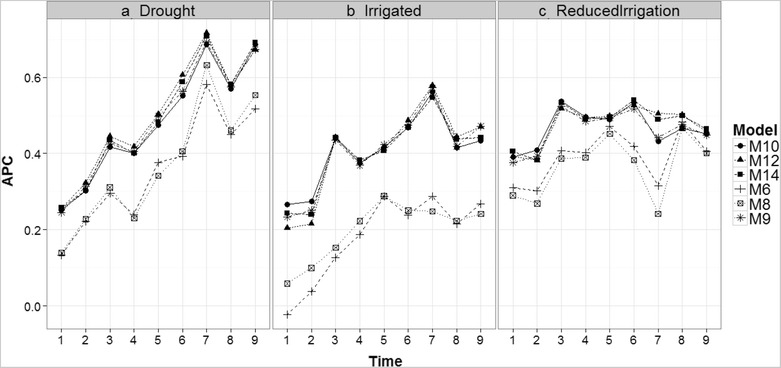
Comparison of time-points in the Drought, Irrigated and Reduced Irrigation environments for models M6, M8, M9, M10, M12, and M14

For this comparison, we ran each of the proposed models using the whole available data set, once for each model in the BGLR package, and for each model we computed the time needed to complete 30,000 iterations. The time (in minutes) needed to run each of the models is given in Fig. 7. The times for running the proposed models are different because the implemented models have different levels of complexity. For this reason, when comparing the models in terms of computational speed measured in minutes, we compared only models with similar levels of complexity.

For example, models M1, M3, M5, M6, M9, M11, and M12 without the $GE_{ij}$ interaction term were, on average, 3.81 times faster than models M2, M4, M7, M8, M10, M13, and M14, which do include this interaction term. Next we compared models M3, M5 and M6 that have the same level of complexity and we observed that functional regression models M5 (254.87 min) and M6 (247.12 min) were faster than model M3 (282.14 min), which is a conventional model. Comparing model M4 versus M7 and M8, we observed that functional models M7 (778.94 min) and M8 (705.63 min) were also faster than conventional model M4 (980.49 min). The same behavior was observed when comparing conventional model M9 (365.69 min) versus functional regression models M11 (274.79 min) and M12 (278.83 min), and conventional model M10 (980.09 min) versus functional regression models M13 (759.70 min) and M14 (712.85 min) (Fig. 7).

### Prediction accuracy of 90% of the lines in one environment using models M13 and M14

Table 3 shows the prediction accuracy of models M11 and M13 for the three environments and all 9 time-points for the 90% of lines that are missing in one environment and predicted using the data in the other two environments (90CV). For time-points 1–5 and 8, the best predictions were for the Irrigated environment, the second best for the Drought environment and the worst for the Reduced Irrigation environment. For time-points 6, 7 and 9, the best predictions were observed in the Drought environment and the worst in the Reduced Irrigation environment.

**Table 3:** Prediction accuracy (average of the ten trn–tst random partitions of the Pearson correlation, APC) of models M11 and M13 for time-points 1–9 for each environment for 90CV when 90% of lines are missing in only one environment (standard error, SE)

Time-point	Drought		Irrigated		Reduced irrigation	
	APC	SE	APC	SE	APC	SE
M11						
1	0.142	0.017	0.244	0.019	0.175	0.013
2	0.197	0.016	0.231	0.027	0.145	0.019
3	0.307	0.018	0.422	0.013	0.315	0.022
4	0.248	0.014	0.347	0.020	0.238	0.019
5	0.298	0.016	0.394	0.016	0.259	0.015
6	0.415	0.006	0.459	0.011	0.310	0.022
7	0.589	0.008	0.528	0.010	0.257	0.020
8	0.422	0.009	0.411	0.014	0.198	0.024
9	0.604	0.009	0.422	0.007	0.313	0.020
M13						
1	0.166	0.015	0.262	0.016	0.216	0.014
2	0.202	0.016	0.234	0.027	0.163	0.017
3	0.307	0.015	0.416	0.017	0.328	0.020
4	0.245	0.015	0.339	0.021	0.238	0.013
5	0.314	0.015	0.409	0.011	0.277	0.016
6	0.427	0.008	0.456	0.013	0.347	0.013
7	0.598	0.011	0.531	0.011	0.280	0.025
8	0.416	0.010	0.402	0.018	0.171	0.021
9	0.613	0.015	0.416	0.006	0.354	0.018

It is interesting to point out that for time-point 3, the models had relatively high prediction accuracy of the unobserved 90% of the phenotypes of lines in one environment (90CV). When the predictions of M11 and M13 are compared, the predictions are very similar under both models (M11 and M13). The results of Table 3 for models M11 and M13 take into account the genomic information (WG), but similar results were obtained with pedigree information (WA) and without pedigree or genomic information (WO).

## Discussion

In this paper, we propose models with main and interaction terms for analyzing HTPP in wheat trial data that take into account genomic and pedigree information. Some of the proposed models take into account G × E or B × E, or both. We found that the models that take into account genomic or pedigree information are similar to the models that ignore this information. For this reason, in this particular study adding pedigree or genomic information did not help to increase prediction accuracy. However, we are aware that in other sets of data the gain by including this information can be very helpful. But the most important issue here is that our proposed method is able to jointly model pedigree or genomic information with hyper-spectral information and we provide R code that is very easy for the implementation. Also, we found that including G × E interaction did not increase prediction accuracy, since not all the models that take this term into account were better in terms of prediction accuracy than those that do not include it. This result might be because the variability among the three environments, Drought, Irrigated and Reduced Irrigation, was only due to the level of irrigation. However, all the models that include B × E interaction had better prediction accuracy than models that do not. Therefore, our results support our initial hypothesis that when HTPP data are collected in multiple environments, it is important to take into account the interaction terms because they can help increase prediction accuracy. However, the magnitude of the increase in prediction accuracy depends on the magnitude of the interaction terms, that is, on the strength of the variation in the phenotype of the same lines between environments. We also found that in the three environments, the lower the time-point, the worse the predictions. However, in the Reduced Irrigation environment, the best predictions were observed at time-point 6, while in the Drought and Irrigated environment, the best predictions were observed at time-point 7. On the other hand, when we wanted to predict 90% of the lines that were unobserved in only one environment, the best predictions were observed at time-points 7 and 9 in two environments (Drought and Irrigated), but even at time-point 6, reasonable predictions were observed for these two environments. In general, there is an optimum time-point that has the best prediction ability. In this study the three environments were considered as specific ecological conditions (represeting target populations of environments) with one environment per ecological condition, therefore further studies including sample of more environments per ecological conditions will be necessary for further investigation.

It is important to point out that models M3, M5 and M6 are equivalent to the single-environment models with bands proposed by Montesinos-López et al. [13], and comparing these models with the rest of the models we see that models M3, M5 and M6 are only superior to models M1, M2 which ignore the information of the bands. But in general these models (M3, M5 and M6) produce lower prediction accuracies than the rest of the models proposed here (M7–M14). This provides empirical evidence that taking into account mainly the band x environment interaction term helps to improve prediction accuracy.

### Bayesian functional regression models

Another important result of this study is that in addition to conventional models (M1, M2, M3, M4, M9, and M10), we proposed functional regression models (M5, M6, M7, M8, M11, M12, M13, and M14). The Bayesian functional regression is an emerging statistical approach that is useful when hundreds of variables are repeatedly measured in each experimental unit, yielding a large number of observations. The primary observation unit is viewed as a curve or, usually, a function (such as in the context of HTPP data, where hundreds of data points are measured at different wavelengths for each unit). This characteristic of the data complicates the use of standard longitudinal modeling strategies, such as random effect models and marginal models, where rigorous assumptions of intra-subject correlation structure are required. Functional regression analysis is increasing in popularity because few assumptions are required for the mean structures and no assumptions are needed for the intra-unit correlation structure of the data. Under this approach, observations of the same unit are viewed as a sample from a functional space, that is, the discrete samples measured are assumed to come from an underlying curve with continuous function forms.

The proposed functional regression models (with B-spline and Fourier basis) turned out to be as competitive as conventional regression models, but the functional regression models, as compared to conventional models, have the advantage of being parsimonious because fewer beta coefficients are needed. For example, in our study, instead of 250 beta coefficients needed for modeling the 250 bands, only 21 of them (that correspond to 21 basis) were used to represent each curve. In terms of implementation speed, the functional regression models only reduced the required time around 22%, on average, when compared to the conventional models. However, as the number of bands increases (>1000), the speed of the functional regression models should be considerably faster than that of the conventional models, since we should be able to model hundreds of bands with only a few bases. It can be hypothetized that the 250 functional predictors could be treated in the same way as usually SNP markers are treated in GBLUP method, that is, to build a band relationship matrix with the 250 wavelengths. In this case the model will include another kernel (the band kernel) and it will be essentially equivalent in terms of complexity to that used in GBLUP even if the number of bands is very large. Further research should be performed to compare the prediction accuracy of a band relationship model versus that achieved using the functional regression models.

### Implementation of the Bayesian functional regression models

Our study’s third significant contribution is that the proposed functional regression models can be implemented with the existing software. We implemented these models using the Bayesian Generalized Linear Regression (BGLR) R-package [5]. Details of the implementation of the proposed models (conventional and functional regression) are given in “Appendix 1”. Implementing the functional regression models in the standard software is possible because a two-step process is needed to model a functional regression model. In the first stage, the corresponding design matrix for the functional covariate is computed; in the second stage, this design matrix is assumed known, so the required parameters that include the parameters for the functional covariate are estimated. The steps required to implement and use the design matrix in all functional regressions models are given in “Appendix 1”. First, the design matrix for the functional covariate(s) is created; then this design matrix is used to estimate the parameters of the functional beta coefficients.

### Advantages of the Bayesian functional regression models

The proposed approach for implementing functional regression models is flexible, as it can be implemented with complex models and large data sets. This is not possible with the ‘fda.usc’ library of Febrero Bande and Oviedo de la Fuente [7], which was created for implementing functional data analysis (exploratory, descriptive, regression analysis of functional data) and recently used by Montesinos-López et al. [13] for single-environment models. Another advantage of implementing our proposed functional regression models in BGLR is that we can change the priors for the random or fixed effects of the functional covariates, which makes it possible to implement variable selection ideas and the Bayesian alphabet (Bayesian ridge regression, Bayes A, Bayes B, Bayes C, Bayes Lasso and GBLUP) in a straightforward manner. As already mentioned, Bayesian Functional regression models are parsimonious, so that as the number of bands increases, the computing time of these models provides an important advantage over conventional models.

## Conclusions

In this paper, we propose conventional Bayesian regression models and Bayesian functional regression models for jointly modelling HTPP data with pedigree or genomic information that take into account interaction terms (G × E and B × E). We found that, in this case, taking into account genomic (or pedigree) information did not improve prediction accuracy in comparison to those models that ignore this information, but the proposed method are easy to implement in R under a Bayesian framework. We also found that G × E did not improve the models’ prediction accuracy, but B × E interaction did. We also found that in general, in the three environments, the lower the time-point, the worse the predictions, while in the Reduced Irrigation environment, the best predictions were observed at time-point 6. In the 90CV, when 90% of the lines in one environment are predicted using information from the other environments, the best predictions were for time-points 7 and 9 in the three environments. The proposed Bayesian functional regression models (with B-splines and Fourier basis) were implemented in R-software BGLR, which is very popular in genomic selection for whole-genome prediction.

We also provide details for implementing the proposed models and those familiar with the BGLR package will realize that implementing the two Bayesian functional regressions models with B-spline and Fourier basis is straightforward. In terms of prediction accuracy, the proposed functional regression models (with B-splines and Fourier basis) were not better than the conventional regression models; however, the functional regression models were slightly better than the conventional models in terms of computational speed since the functional regression models were slightly faster than the conventional models. This was due to the reduced number of beta coefficients that need to be estimated for the functional regression models compared to those needed in the conventional models. We found that the best models in terms of prediction accuracy were those that take into account B × E interaction.

## Appendix 2: Additional plots

See Figs. 8, 9, 10, 11, and 12.

## Appendix 3

Standard errors (SE) fof the average Pearson correlations (APC) for each proposed model, time point, environment, without genomic and pedigree information (WO), only with pedigree information (A), and only with genomic information (WG).

**Table:**

Model	Time	WO			WA			WG		
		Drought	Irrigated	Reduced irrigation	Drought	Irrigated	Reduced irrigation	Drought	Irrigated	Reduced irrigation
		SE	SE	SE	SE	SE	SE	SE	SE	SE
M1	1	0.014	0.012	0.02	0.013	0.013	0.02	0.014	0.012	0.018
M1	2	0.014	0.012	0.02	0.013	0.013	0.02	0.014	0.012	0.018
M1	3	0.014	0.012	0.02	0.013	0.013	0.02	0.014	0.012	0.018
M1	4	0.014	0.012	0.02	0.013	0.013	0.02	0.014	0.012	0.018
M1	5	0.014	0.012	0.02	0.013	0.013	0.02	0.014	0.012	0.018
M1	6	0.014	0.012	0.02	0.013	0.013	0.02	0.014	0.012	0.018
M1	7	0.014	0.012	0.02	0.013	0.013	0.02	0.014	0.012	0.018
M1	8	0.014	0.012	0.02	0.013	0.013	0.02	0.014	0.012	0.018
M1	9	0.014	0.012	0.02	0.013	0.013	0.02	0.014	0.012	0.018
M2	1	0.011	0.011	0.02	0.023	0.027	0.019	0.013	0.015	0.019
M2	2	0.011	0.011	0.021	0.012	0.023	0.017	0.012	0.013	0.017
M2	3	0.011	0.011	0.021	0.021	0.018	0.017	0.008	0.01	0.015
M2	4	0.012	0.011	0.021	0.022	0.026	0.018	0.008	0.009	0.016
M2	5	0.011	0.011	0.022	0.008	0.014	0.016	0.006	0.013	0.015
M2	6	0.012	0.011	0.021	0.012	0.015	0.018	0.009	0.01	0.013
M2	7	0.012	0.012	0.02	0.016	0.02	0.017	0.008	0.011	0.009
M2	8	0.011	0.011	0.02	0.011	0.016	0.017	0.011	0.012	0.012
M2	9	0.011	0.011	0.02	0.012	0.019	0.015	0.009	0.011	0.011
M3	1	0.014	0.016	0.019	0.011	0.016	0.019	0.013	0.014	0.018
M3	2	0.014	0.013	0.017	0.014	0.012	0.018	0.014	0.012	0.016
M3	3	0.012	0.011	0.016	0.01	0.012	0.016	0.011	0.008	0.014
M3	4	0.013	0.014	0.013	0.011	0.013	0.013	0.012	0.011	0.012
M3	5	0.013	0.011	0.016	0.011	0.01	0.017	0.012	0.009	0.015
M3	6	0.015	0.015	0.019	0.014	0.016	0.017	0.013	0.014	0.018
M3	7	0.011	0.015	0.013	0.009	0.016	0.014	0.009	0.013	0.011
M3	8	0.008	0.012	0.017	0.007	0.01	0.018	0.009	0.009	0.015
M3	9	0.011	0.009	0.014	0.009	0.009	0.015	0.009	0.008	0.013
M4	1	0.013	0.018	0.02	0.008	0.018	0.017	0.01	0.017	0.013
M4	2	0.013	0.015	0.016	0.017	0.015	0.013	0.01	0.01	0.01
M4	3	0.01	0.014	0.013	0.009	0.019	0.017	0.011	0.012	0.017
M4	4	0.013	0.015	0.015	0.005	0.019	0.013	0.01	0.004	0.015
M4	5	0.011	0.013	0.013	0.007	0.014	0.015	0.015	0.008	0.017
M4	6	0.017	0.015	0.018	0.011	0.014	0.016	0.013	0.008	0.015
M4	7	0.007	0.013	0.009	0.013	0.014	0.009	0.007	0.016	0.01
M4	8	0.008	0.011	0.013	0.01	0.012	0.016	0.007	0.01	0.01
M4	9	0.011	0.009	0.01	0.007	0.009	0.012	0.008	0.01	0.013
M5	1	0.014	0.012	0.018	0.012	0.013	0.019	0.014	0.012	0.017
M5	2	0.013	0.011	0.021	0.012	0.012	0.021	0.012	0.011	0.019
M5	3	0.012	0.011	0.018	0.01	0.012	0.019	0.01	0.01	0.016
M5	4	0.013	0.014	0.016	0.011	0.014	0.016	0.012	0.012	0.014
M5	5	0.014	0.013	0.016	0.011	0.014	0.018	0.012	0.013	0.015
M5	6	0.014	0.015	0.02	0.01	0.018	0.02	0.012	0.016	0.018
M5	7	0.012	0.017	0.012	0.009	0.016	0.012	0.01	0.016	0.011
M5	8	0.011	0.014	0.017	0.009	0.013	0.017	0.011	0.01	0.015
M5	9	0.012	0.008	0.015	0.008	0.01	0.016	0.008	0.011	0.014
M6	1	0.014	0.013	0.018	0.012	0.015	0.018	0.013	0.013	0.016
M6	2	0.014	0.011	0.021	0.012	0.012	0.021	0.013	0.01	0.018
M6	3	0.012	0.012	0.018	0.009	0.013	0.018	0.011	0.012	0.016
M6	4	0.013	0.013	0.016	0.011	0.014	0.015	0.012	0.01	0.013
M6	5	0.013	0.013	0.015	0.01	0.014	0.017	0.012	0.013	0.015
M6	6	0.012	0.012	0.019	0.01	0.011	0.019	0.012	0.01	0.017
M6	7	0.01	0.015	0.011	0.008	0.016	0.013	0.009	0.015	0.01
M6	8	0.011	0.011	0.016	0.009	0.011	0.017	0.009	0.009	0.015
M6	9	0.011	0.008	0.015	0.008	0.009	0.016	0.01	0.008	0.013
M7	1	0.014	0.018	0.02	0.018	0.026	0.02	0.012	0.016	0.018
M7	2	0.01	0.013	0.019	0.008	0.019	0.018	0.008	0.016	0.015
M7	3	0.011	0.016	0.014	0.009	0.02	0.014	0.011	0.012	0.015
M7	4	0.013	0.022	0.015	0.013	0.02	0.015	0.009	0.008	0.012
M7	5	0.013	0.014	0.015	0.01	0.014	0.016	0.009	0.009	0.014
M7	6	0.017	0.015	0.019	0.013	0.017	0.016	0.009	0.011	0.012
M7	7	0.008	0.013	0.012	0.017	0.011	0.016	0.009	0.011	0.023
M7	8	0.01	0.009	0.014	0.008	0.012	0.015	0.011	0.008	0.015
M7	9	0.01	0.007	0.011	0.01	0.008	0.012	0.01	0.011	0.013
M8	1	0.015	0.017	0.02	0.014	0.026	0.017	0.009	0.016	0.016
M8	2	0.012	0.013	0.019	0.016	0.015	0.021	0.008	0.011	0.013
M8	3	0.012	0.015	0.014	0.007	0.018	0.015	0.01	0.014	0.016
M8	4	0.011	0.014	0.013	0.008	0.014	0.014	0.011	0.011	0.01
M8	5	0.011	0.01	0.012	0.005	0.01	0.016	0.012	0.009	0.014
M8	6	0.013	0.007	0.014	0.018	0.012	0.016	0.012	0.008	0.013
M8	7	0.007	0.011	0.009	0.011	0.011	0.015	0.009	0.014	0.02
M8	8	0.008	0.009	0.013	0.007	0.011	0.017	0.01	0.01	0.014
M8	9	0.01	0.008	0.011	0.01	0.01	0.012	0.009	0.011	0.012
M9	1	0.011	0.011	0.02	0.009	0.011	0.021	0.01	0.01	0.019
M9	2	0.01	0.01	0.014	0.01	0.011	0.015	0.01	0.01	0.012
M9	3	0.008	0.01	0.013	0.005	0.011	0.013	0.008	0.01	0.011
M9	4	0.01	0.009	0.012	0.008	0.007	0.013	0.01	0.006	0.009
M9	5	0.008	0.007	0.013	0.006	0.005	0.013	0.008	0.004	0.011
M9	6	0.007	0.007	0.015	0.006	0.007	0.015	0.007	0.008	0.011
M9	7	0.007	0.008	0.011	0.006	0.008	0.013	0.006	0.008	0.007
M9	8	0.006	0.008	0.014	0.005	0.008	0.012	0.004	0.007	0.01
M9	9	0.008	0.011	0.012	0.005	0.011	0.013	0.006	0.012	0.01
M10	1	0.011	0.007	0.012	0.011	0.008	0.017	0.013	0.01	0.015
M10	2	0.009	0.006	0.012	0.013	0.013	0.014	0.01	0.007	0.012
M10	3	0.008	0.009	0.01	0.008	0.009	0.013	0.007	0.009	0.012
M10	4	0.009	0.008	0.011	0.006	0.006	0.014	0.008	0.008	0.009
M10	5	0.008	0.008	0.014	0.006	0.007	0.013	0.011	0.005	0.008
M10	6	0.006	0.009	0.01	0.004	0.006	0.015	0.008	0.007	0.011
M10	7	0.007	0.01	0.008	0.006	0.01	0.013	0.007	0.009	0.009
M10	8	0.007	0.008	0.017	0.006	0.006	0.013	0.004	0.006	0.007
M10	9	0.008	0.013	0.007	0.009	0.009	0.013	0.007	0.015	0.009
M11	1	0.01	0.012	0.018	0.008	0.014	0.019	0.01	0.012	0.016
M11	2	0.01	0.01	0.018	0.009	0.011	0.017	0.01	0.008	0.015
M11	3	0.009	0.009	0.015	0.007	0.009	0.015	0.009	0.008	0.014
M11	4	0.011	0.008	0.015	0.008	0.008	0.016	0.011	0.008	0.013
M11	5	0.009	0.01	0.015	0.007	0.008	0.014	0.007	0.008	0.012
M11	6	0.007	0.009	0.017	0.006	0.009	0.017	0.006	0.009	0.014
M11	7	0.006	0.008	0.009	0.006	0.009	0.013	0.005	0.009	0.009
M11	8	0.006	0.007	0.013	0.005	0.007	0.014	0.004	0.006	0.011
M11	9	0.007	0.013	0.013	0.006	0.012	0.013	0.007	0.014	0.01
M12	1	0.01	0.012	0.019	0.008	0.014	0.02	0.009	0.012	0.017
M12	2	0.012	0.01	0.018	0.009	0.011	0.017	0.01	0.01	0.015
M12	3	0.011	0.012	0.015	0.01	0.011	0.016	0.011	0.01	0.015
M12	4	0.011	0.011	0.014	0.009	0.01	0.015	0.012	0.011	0.013
M12	5	0.011	0.011	0.017	0.009	0.009	0.018	0.007	0.008	0.013
M12	6	0.008	0.008	0.016	0.005	0.009	0.016	0.006	0.009	0.015
M12	7	0.006	0.008	0.009	0.005	0.009	0.011	0.007	0.009	0.008
M12	8	0.007	0.008	0.014	0.005	0.008	0.013	0.006	0.008	0.012
M12	9	0.007	0.013	0.012	0.006	0.013	0.014	0.007	0.014	0.01
M13	1	0.012	0.009	0.013	0.012	0.018	0.017	0.008	0.01	0.014
M13	2	0.008	0.008	0.014	0.007	0.018	0.015	0.007	0.007	0.015
M13	3	0.009	0.008	0.01	0.006	0.01	0.013	0.007	0.007	0.008
M13	4	0.009	0.008	0.012	0.006	0.007	0.013	0.007	0.006	0.01
M13	5	0.008	0.007	0.014	0.006	0.01	0.013	0.008	0.006	0.008
M13	6	0.006	0.009	0.01	0.006	0.008	0.012	0.012	0.008	0.007
M13	7	0.006	0.01	0.007	0.006	0.008	0.013	0.009	0.012	0.009
M13	8	0.006	0.007	0.015	0.007	0.006	0.014	0.007	0.005	0.013
M13	9	0.006	0.015	0.008	0.01	0.013	0.014	0.006	0.013	0.01
M14	1	0.009	0.008	0.013	0.012	0.015	0.016	0.01	0.007	0.014
M14	2	0.009	0.008	0.014	0.008	0.015	0.016	0.008	0.009	0.011
M14	3	0.011	0.009	0.011	0.009	0.008	0.015	0.01	0.009	0.012
M14	4	0.011	0.01	0.014	0.009	0.013	0.012	0.011	0.009	0.009
M14	5	0.008	0.009	0.014	0.01	0.008	0.016	0.009	0.007	0.011
M14	6	0.006	0.009	0.011	0.004	0.005	0.014	0.01	0.009	0.009
M14	7	0.008	0.008	0.006	0.006	0.009	0.01	0.009	0.013	0.012
M14	8	0.006	0.007	0.012	0.005	0.01	0.014	0.005	0.01	0.009
M14	9	0.008	0.014	0.008	0.009	0.012	0.012	0.007	0.012	0.006
